# Eighteenth-century genomes show that mixed infections were common at time of peak tuberculosis in Europe

**DOI:** 10.1038/ncomms7717

**Published:** 2015-04-07

**Authors:** Gemma L. Kay, Martin J. Sergeant, Zhemin Zhou, Jacqueline Z.-M. Chan, Andrew Millard, Joshua Quick, Ildikó Szikossy, Ildikó Pap, Mark Spigelman, Nicholas J. Loman, Mark Achtman, Helen D. Donoghue, Mark J. Pallen

**Affiliations:** 1 Microbiology and Infection Unit, Division of Translational and Systems Medicine, Warwick Medical School, University of Warwick, Gibbet Hill Road, Coventry CV4 7AL, UK; 2 Institute of Microbiology and Infection, School of Biosciences, University of Birmingham, Birmingham B15 2TT, UK; 3 Department of Anthropology, Hungarian Natural History Museum, Ludovika tér 2–6, 1083 Budapest, Hungary; 4 Department of Anatomy and Anthropology, Sackler Faculty of Medicine, Tel-Aviv University, Tel-Aviv 9112102, Israel; 5 Centre for Clinical Microbiology, Division of Infection and Immunity, University College London, London NW3 2PF, UK

## Abstract

Tuberculosis (TB) was once a major killer in Europe, but it is unclear how the strains and patterns of infection at ‘peak TB' relate to what we see today. Here we describe 14 genome sequences of *M. tuberculosis*, representing 12 distinct genotypes, obtained from human remains from eighteenth-century Hungary using metagenomics. All our historic genotypes belong to *M. tuberculosis* Lineage 4. Bayesian phylogenetic dating, based on samples with well-documented dates, places the most recent common ancestor of this lineage in the late Roman period. We find that most bodies yielded more than one *M. tuberculosis* genotype and we document an intimate epidemiological link between infections in two long-dead individuals. Our results suggest that metagenomic approaches usefully inform detection and characterization of historical and contemporary infections.

## Abstract (web summary)

Tuberculosis was once a major killer in Europe. Here the authors use metagenomics to obtain genomic sequences of *Mycobacterium tuberculosis* from human remains from eighteenth-century Hungary, revealing mixed infections within individuals as well as presence of the same strain in two individuals.

## Graphical abstract

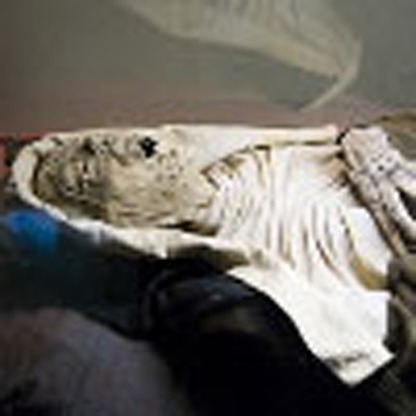

Tuberculosis (TB), although still a major global health problem, was once much more common in Europe than it is today—for example, when first describing the bacterial aetiology of TB in 1882, Robert Koch claimed that this infection accounted for one in seven deaths^1^. However, it remains unclear when TB reached peak prevalence in Europe and how the epidemiology of infection differed in a historical high-prevalence context from what we see today. In addition, dates of origin of key TB lineages remain contentious: for example, recent estimates of the *M. tuberculosis* complex, which contains human- and animal-associated lineages, vary by an order of magnitude from 70,000 to <6000 years ago^2^,^3^.

Here, we address these questions by analysing 14 historical genome sequences of *M. tuberculosis* with well-documented dates, obtained from human remains from eighteenth-century Hungary using shotgun metagenomics (direct sequencing of DNA from samples without target-specific capture or amplification)^4^,^5^. Our samples originate from a crypt in the Dominican church of Vác in Hungary (Fig. 1) that was used to house the remains of affluent Catholics during the eighteenth and early nineteenth centuries. When re-discovered in 1994, it was found to contain the remains of over 200 individuals. Most of these had undergone natural mummification and for many, names and dates of death were available from written records. Previous pathological and molecular investigations showed that around half those sampled were infected with TB^6^ and, in a preliminary analysis, some of us showed that genomic data could be acquired from one Vác sample^5^.

In this study, we show that all the historic genotypes from Vác belong to *M. tuberculosis* Lineage 4. Bayesian phylogenetic dating places the most recent common ancestor of this lineage in the late Roman period. We find that most bodies yielded more than one *M. tuberculosis* genotype and we document an intimate epidemiological link between infections in two long-dead individuals.

## Results

### Genome sequences

**Figure 2:**
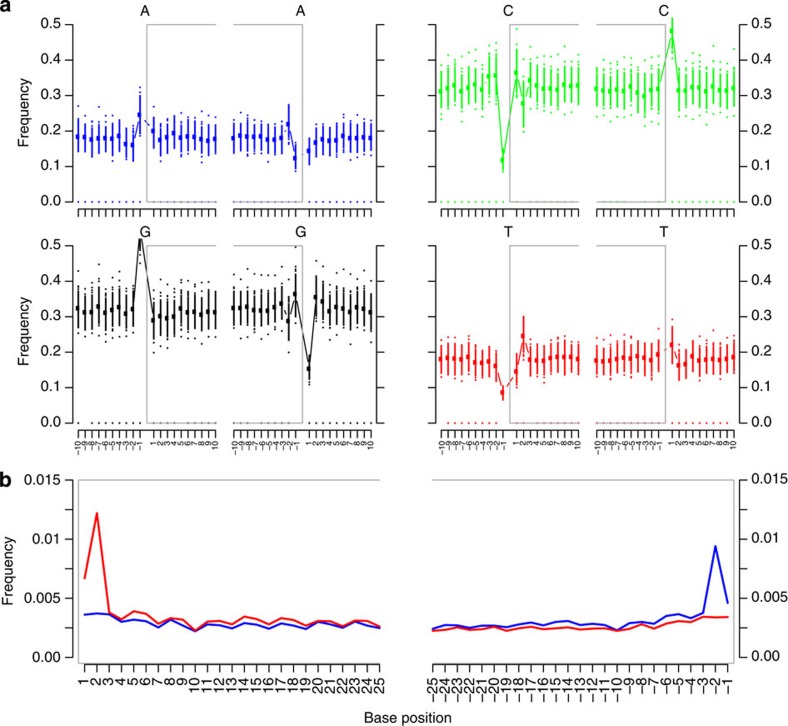
Signatures of DNA damage associated with aged DNA. These data from body 28 are representative of all eighteenth-century samples. (**a**) The four panels show the average base frequencies at positions within individual reads (grey box) flanked by all calls from reads in neighbouring sequences. (**b**) Frequencies of specific base substitutions at specific positions near the 5′-end (left panel) and 3′-end (right panel) occurring within reads. C to T changes are indicated by a red line, and G to A changes by a blue line.

We extracted DNA from samples from 26 bodies from the Vác crypt with previous evidence of infection with TB (Table 1 and Supplementary Table 1). We converted the DNA into Illumina libraries, which were then sequenced alongside three blank controls. Sequencing reads were then mapped to the reference genome of *M. tuberculosis* strain H37Rv (Genbank accession code NC_000962.2) under conditions stringent enough to exclude spurious hits to conserved genes from related environmental organisms (<3 mismatches per 100 bases; exclusion of reads mapping to rRNA genes). In this way, we obtained draft *M. tuberculosis* genome sequences from eight bodies (Table 1). From five of the eight bodies we recovered multiple *M. tuberculosis* genome sequences (Supplementary Figs 1 and 2), so that, in total, we acquired 14 eighteenth-century *M. tuberculosis* genome sequences, 4 of them at >10X coverage (B68–1, B68-2, B80, B92-1). No significant matches to *M. tuberculosis* were found in the negative controls. Among the historical *M. tuberculosis* reads, we found a bias for a purine before the start of reads, consistent with the depurination seen in aged DNA, although, as with medieval leprosy^7^, some signatures of ancient DNA damage were absent, including CT and GA base conversions at the 5′ and 3′ ends (Fig. 2).

### Phylogenetic analyses

**Figure 3:**
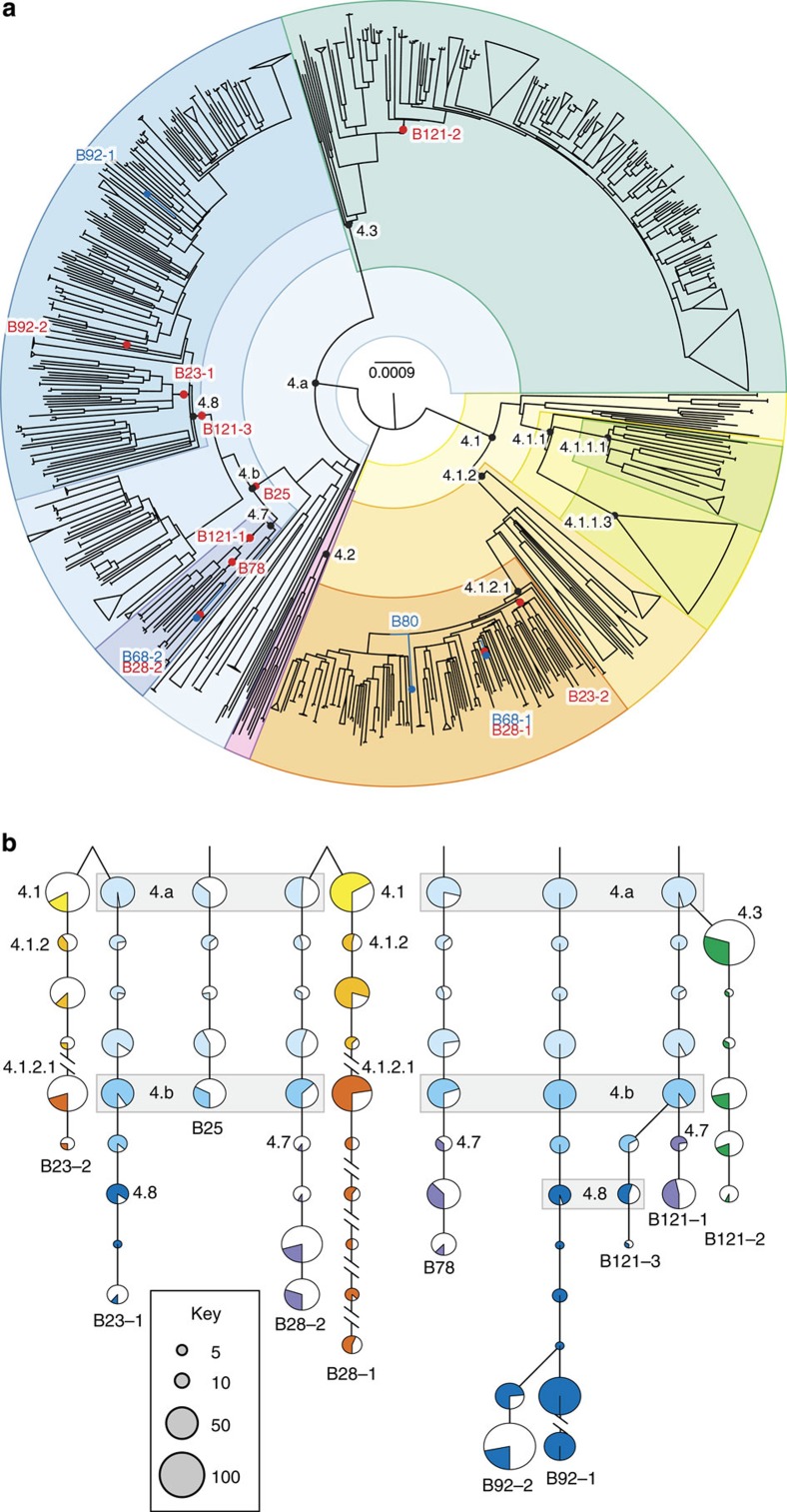
Phylogeny of modern and eighteenth-century *M. tuberculosis* Lineage 4 genotypes. (**a**) Maximum-likelihood tree of 1,582 modern Lineage 4 genotypes and four high-coverage historical genotypes (B68-1, B68-2, B80 and B92-1; blue lines with dots at the tips; details in Supplementary Data 2). The tree was rooted using a Beijing genotype (not shown). Ten additional low-coverage historical genotypes were mapped to the tree with MGplacer (red nodes). Lineages and sub-lineages used for dating (Supplementary Table 3) are indicated by black nodes and coloured segments. (**b**) Topological representation of phylogenetic paths through nodes outwards from the root (top) for ten low-coverage historical genotypes; pie-charts show SNVs recovered per node (coloured segment) from each low-coverage genome as a proportion of all polymorphic sites defining that node according to MGplacer.

In all 14 historical genomes, we detected a seven base-pair deletion in the *pks15/1* gene, characteristic of the Euro-American lineage of *M. tuberculosis*^8^,^9^. This lineage, also termed Lineage 4, currently accounts for over a million TB cases a year in Europe and in the Americas^10^. To determine precise relationships between historical and modern strains, we retrieved 1,582 contemporary unassembled Lineage 4 genomes drawn from four collections^11^,^12^,^13^,^14^ and one Beijing-lineage genome as outgroup. We mapped reads from the contemporary genomes and from our four high-coverage historical genomes against the H37Rv reference genome. We then generated a phylogenetic tree from single-nucleotide variants (SNVs) found in non-repetitive regions and adapted a recently described hierarchical nomenclature^15^ to define nodes and sub-clades within the tree (Fig. 3a and Supplementary Data 1–3). In this way, we established that our four high-coverage historical genomes belonged to phylogenetically distinct genotypes (Fig. 3a and Table 1).

Conventional phylogenetic methods that rely on identification of all trusted SNVs within a genome could not be applied to the ten low-coverage genome sequences we obtained. We thus adapted the technique of phylogenetic placement, whereby low-coverage genomes are placed on a fixed reference tree, computed from high-coverage genomes. To do this, we reconstructed the sequences of all nodes within the Lineage 4 phylogeny and documented the SNVs that characterized each node. We then devised a new algorithm, MGplacer, capable of mapping low-coverage genomes, including those from multiple-genotype samples, to successive nodes within the tree. This approach allowed us to perform phylogenetic placements for all ten low-coverage historical genomes (Fig. 3). These phylogenetic analyses revealed that there were at least 12 distinct strains of *M. tuberculosis* circulating in eighteenth-century Hungary. This means we can rule out a clonal outbreak caused by a single particularly virulent strain as the explanation for the high prevalence of TB in this population. Furthermore, the deep nesting of our historical *M. tuberculosis* genotypes within contemporary sub-divisions of the Lineage 4 phylogeny confirms continuity of strain lineages over the past two centuries.

### Dating

**Figure 4:**
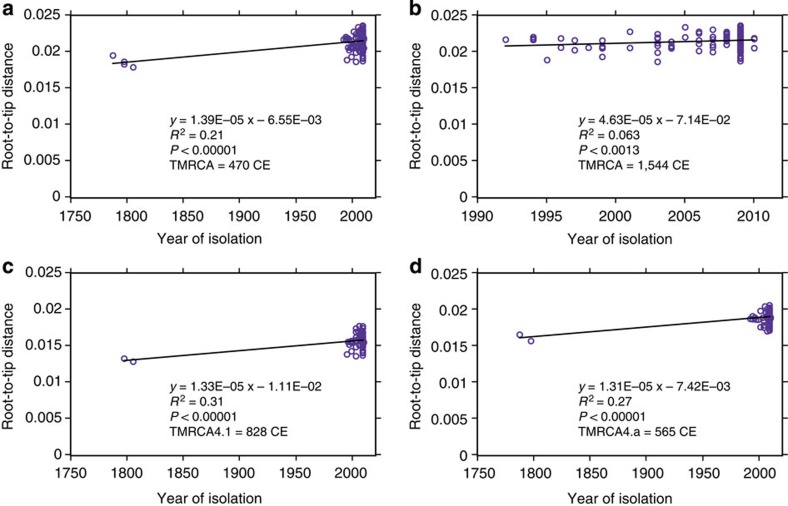
Linear regression plots of root-to-tip distances according to Path-O-Gen (http://tree.bio.ed.ac.uk/software/pathogen/). (**a**) All 165 genotypes in Lineage 4, including four high-coverage eighteenth-century genomes. (**b**) Only the 161 modern isolates. (**c**) Genotypes from sub-lineage 4.1. (**d**) Genotypes from sub-lineage 4.a.

**Figure 5:**
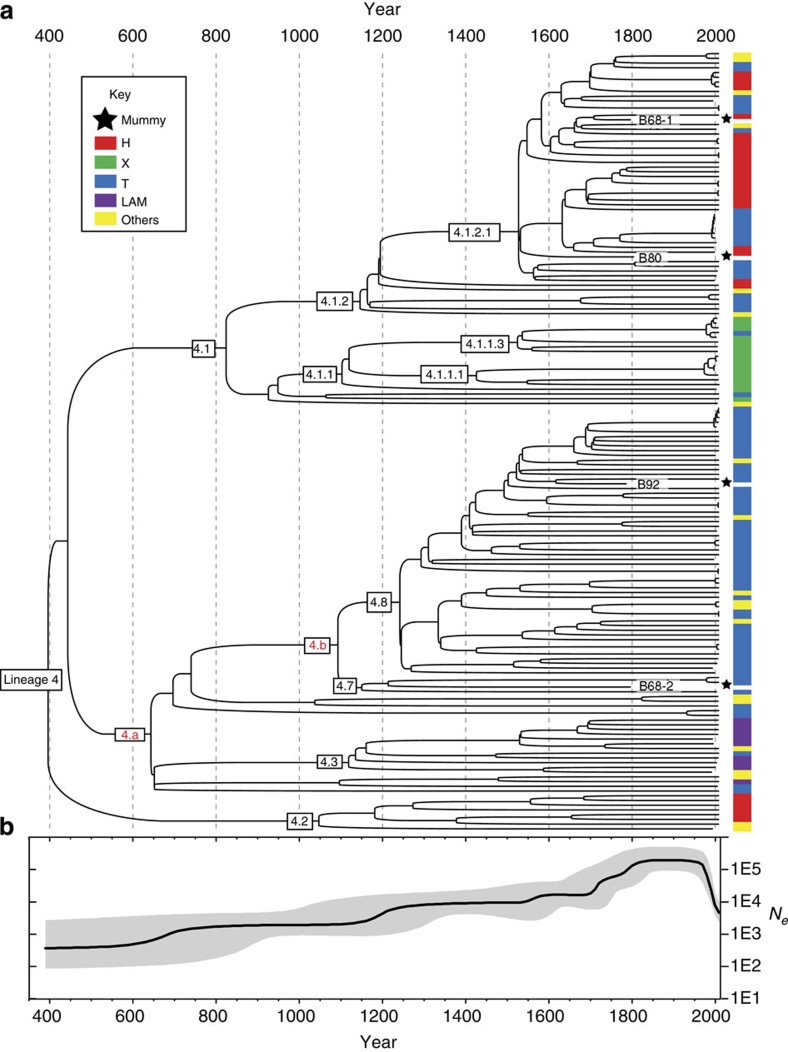
Bayesian phylogeny and population dynamics of 165 genotypes from Lineage 4, calibrated with four high-coverage eighteenth-century genotypes. SNPs in the non-repetitive core genome (Supplementary Data 1) were analysed with BEAST^17^ using UCLD clock rate and a Bayesian Skyline with 30 steps (details in Supplementary Table 4). (**a**) Maximum clade credibility tree with nodes (boxes) labelled according to the hierarchical nomenclature of Coll *et al*.^15^, with two additional nodes 4.a and 4.b. Supplementary Table 2 summarizes the dating estimates for nodes. Short branches corresponding to four historical genotypes are labelled by name and highlighted by asterisks. Coloured boxes show broad spoligotype groupings for modern isolates, illustrating the paraphyletic nature of these groups (details in Supplementary Fig. 3). (**b**) Bayesian skyline plot showing changes over time in effective population size, *N*_e_ (in black) since 396 CE, with 95% confidence intervals in grey.

Next, we estimated divergence times for Lineage 4 and its sub-lineages. For dating, we selected the four high-coverage historical genomes, which had well-documented dates of death for tip calibration, as well as 161 modern genomes from Lineage 4 that spanned a range of isolation dates from 1992 to 2010 (Supplementary Table 2). We detected significant clock-like behaviour for the Lineage 4 tree when data from modern and historical isolates were combined (Fig. 4). We obtained consistent dates of the most recent common ancestors of Lineage 4 and of several internal nodes using two different programmes, Path-O-Gen^16^ and BEAST^17^ (Fig. 5a, Supplementary Table 3; Fig. 4 and Supplementary Fig. 3). The Path-O-Gen analysis reported the date of the most recent common ancestor of Lineage 4 as 470 CE, whereas the median estimate from the best of 12 BEAST models placed the date at 396 CE, that is, within the late Roman period, with a range between models that spanned the Iron Age to the end of Antiquity (Supplementary Table 4). Our dating is consistent with evidence that a strain containing the *pks15/1* deletion was present in Britain by the second–fourth centuries CE^18^.

In line with historical epidemiological records^19^,^20^, a Bayesian Skyline plot^21^ shows that the effective population size for Lineage 4 increased continuously from its origin until the twentieth century, when it underwent a precipitous decline (Fig. 5b). Our median estimate of the mutation rate for Lineage 4 is 5.00 × 10^−08^ substitutions per nucleotide per year (Supplementary Table 4). This mutation rate, calibrated with accurate historical dates, is similar to rates estimated from contemporary *M. tuberculosis* genomes^11^ and from *M. pinnepedii* genomes that were obtained from samples radiocarbon-dated to 1028–1280 CE^3^. This mutation rate is consistent with the hypothesis that the most recent common ancestor of the *M. tuberculosis* complex existed <6,000 years ago^3^, but is inconsistent with the recovery of amplification products indicative of sub-lineages within the complex from Neolithic samples^22^,^23^,^24^. One potential explanation for this discrepancy is that some assumptions underlying phylogenetic dating (for example, of a stable substitution rate) may be erroneous. Alternatively, ancient DNA studies that rely on PCR amplification may have been subject to contamination. The recovery of additional *M. tuberculosis* genomes from well-dated historic and prehistoric samples will be needed to settle this issue, including from the Neolithic samples that have generated the sub-lineage-specific amplification products.

### Mixed infections

Microbiological analyses of samples from contemporary TB patients usually report a single strain of *M. tuberculosis* per patient. By contrast, five of the eight bodies in our study yielded more than one *M. tuberculosis* genotype—remarkably, from one individual we obtained three distinct genotypes (Table 1). Although this predominance of mixed infections almost certainly reflects a real difference between the epidemiology of TB today and in this historical setting, mixed infections are still seen in up to a fifth of cases in high-prevalence areas and four distinct *M. tuberculosis* genotypes have been reported from a single patient^25^,^26^,^27^. We thus suspect that multi-strain infection was common during peak TB in Europe. However, as culture-based TB microbiology appears to be poorly suited to the detection of mixed infections^28^, the approaches we describe here might deliver improvements in diagnosis and management of contemporary infections^29^.

### Within family transmission

Two of the bodies we sampled belonged to a family group: Anna Schöner (body 28) was the mother of Terézia Hausmann (body 68). Our analyses on these bodies provide the first evidence of an intimate epidemiological link between TB infections in two long-dead individuals, supporting mother–child transmission, or vice versa, or infection from a common source. More striking is that we obtained the same two *M. tuberculosis* genotypes, albeit in different proportions, from samples from both bodies (Table 1). It remains unclear whether this shared within-host diversity in mother and daughter stems from multiple episodes of infection or from a single transmission event of more than one strain. These findings add weight to the claim that within-host diversity poses a challenge when attempting to infer the nature and direction of disease transmission^30^. Interestingly, two samples from Terézia Hausmann's lung yielded different proportions of the two genotypes, perhaps suggesting fine-grained spatial heterogeneity in the distribution of strains (Supplementary Fig. 2c).

## Discussion

Here, we have confirmed the remarkably high prevalence of TB within an affluent, urbanized, but largely pre-industrial, Central European population. By showing that historical strains can be accurately mapped to contemporary lineages, we have ruled out, for early modern Europe, the kind of scenario recently proposed for the Americas^3^, that is, wholesale replacement of one major lineage by another (with a different host range and presumed pathogen biology) and have confirmed the genotypic continuity of an infection that has ravaged the heart of Europe since prehistoric times^31^. With TB resurgent in many parts of the world, including Hungary^32^, the struggle to control this ancient infection is far from over. Here, we have shown that metagenomic approaches can document past infections. However, we have also recently shown that metagenomics can identify and characterize pathogens in contemporary samples^29^,^33^, so such approaches might soon also inform current and future infectious disease diagnosis and control.

## Methods

### Sample collection and storage

Samples were collected in 1997 and 1999 using a Stortz endoscope and aseptic technique. Sample site, body number and name were recorded and samples were placed into numbered sterile universal bottles, which were then individually wrapped in plastic bags and stored at 4 °C. Relevant biographic information (for example, age, sex, family name and relationships, date and cause of death) was retrieved from contemporary written records, including text on the coffin and information in death, baptismal and marriage registers.

### Extraction of DNA with library preparation and sequencing

DNA was extracted from mummified tissue following a modification of a published protocol^34^. Mummified tissue (15–20 mg) was added to 400 μl of deproteination solution (0.5 M EDTA pH 8.0 and 20 mg ml^−1^ proteinase K) in a sterile 2 ml screw-capped tube containing a minimum of ten glass beads (1–2 mm diameter). The tubes were mixed twice in a mini-bead beater at top speed for 45 s, then incubated shaking at 56 °C for 48–72 h (or until the samples were fully dispersed).

After deproteination, half the slurry was transferred into sterile 15 ml screw-capped tubes (*N*-phenacythiazolium bromide (PTB–)), with 4.5 ml lysis buffer (5 M guanidine thiocyanate, 0.1 M tris-HCl pH 6.4, 0.2 M EDTA pH 8.0 and Triton X-100). PTB (40 μl) was added to the remaining residual slurry in the 2-ml tube and incubated at 56 °C for 1 h. After incubation, the residual slurry was transferred into sterile 15 ml screw-capped tubes (PTB+), with 4.5 ml lysis buffer. Sample tubes were incubated in a water bath at 56 °C for 48–72 h, subjected to three rounds of snap freezing in liquid nitrogen, followed by thawing in a 56 °C water bath.

Tubes were centrifuged at 2,500 r.p.m. (1,258*g*) for 15 min and the supernatants transferred to sterile 15 ml screw-capped tubes. Freshly mixed silica (20 μl) was added and the samples incubated at room temperature for 1 h on a rotator, centrifuged at 2,500 r.p.m. (1,258*g*) for 15 min, the silica supernatants were either stored at 4 °C (PTB– samples) or discarded (PTB+ samples). The silica pellets were dislodged by vortexing, resuspended in 200 μl wash buffer (5 M guanidine thiocyanate, 0.1 M Tris-HCl, pH 6.4) and transferred to sterile 2 ml screw-capped tubes. Residual silica was washed out with another 100 μl wash buffer. Tubes were centrifuged at 14,000 r.p.m. (20,817*g*) for 1 min to pellet the silica and the supernatant was discarded. Silica pellets were washed by resuspending once in 200 μl wash buffer, twice in 200 μl −20 °C filter-sterilized ethanol and once in 200 μl of −20 °C acetone, centrifuging at 14,000 r.p.m. (20,817*g*) for 1 min and discarding washings. Tubes were drained on clean absorbent paper followed by drying at 56 °C for 1 h. Dried preparations were stored at 4 °C until examined.

Dried silica supernatants were rehydrated with 80 μl of filter sterilized elution buffer (EB), mixed and incubated at 60 °C for 15 min. All samples were centrifuged at 14,000 r.p.m. (20,817*g*) for 1 min, 70 μl of supernatant was transferred to 1.5 ml sterile low binding tubes. To remove residual slurry, the supernatant was centrifuged for a further 1 min at 14,000 r.p.m. (20,817*g*) and 60 μl of supernatant was processed. AMPureXP beads (at room temperature) were added to each sample (36 μl) and mixed by pipetting. All samples were incubated for 10 min at room temperature, placed on the magnetic stand until the sample was clear, 94 μl of supernatant was removed and stored for further processing. The remaining beads were washed twice with 80% ethanol for at least 30 s per wash. Beads were air dried for 5 min on the magnetic stand, resuspended in 32.5 μl EB off the magnetic stand and incubated for 5 min at room temperature. After placing on the magnetic stand 30 μl of supernatant was removed and stored at –20 °C in sterile 1.5 ml low binding tubes (DNA fragments >500 bp). The stored 94 μl supernatant was taken and 200 μl AMPure XP beads added, mixed by pipetting and incubated at room temperature for 10 min. Samples were placed on the magnetic stand until clear and 290 μl of supernatant was discarded. The remaining beads were washed twice with 80% ethanol for at least 30 s per wash. Beads were air dried for 10 min on the magnetic stand, resuspended in 65 μl EB off the magnetic stand and incubated for 10 min at room temperature. After placing on the magnetic stand 62.5 μl of supernatant was transferred to sterile 1.5 ml low binding tubes (DNA fragments <500 bp). Extracted DNA (<500 bp) was quantified using HS dsDNA qubit assay (Life Technologies) as per the manufacturer's instructions (2 μl of sample was quantified).

Extracted DNA was converted into TruSeq Nano libraries for sequencing on an Illumina MiSeq according to the manufacturer's low sample protocol with a few modifications. No fragmentation step or size selection after end repair was carried out due to the nature of ancient DNA. Samples were cleaned after end repair with 200 μl sample-purification beads. dA-tailing and adapter ligation were according to the manufacturer's protocol. DNA fragments were enriched using 15 PCR cycles instead of 8. Libraries were quantified using HS dsDNA qubit assay as per the manufacturer's instructions (2 μl of sample was quantified), then stored at −20 °C until preparation for sequencing on the MiSeq.

Libraries were pooled in equimolar amounts (determined by analysis on an Agilent Bioanalyser 2100 and HS dsDNA qubit assay) and 12 pM sequenced on an Illumina MiSeq platform v2 2 × 250 bp paired end protocol. Body 23, 25, 28 and 121 of 4 nM libraries were pooled and sequenced on an Illumina HiSeq platform (rapid run), TruSeq Rapid SBS kit—HS (200 cycle).

### Preventing *M. tuberculosis* contamination

DNA extraction and library preparation (up until the library amplification step) were carried out in a dedicated pre-PCR laboratory in which no mycobacterial strains had been cultured, no mycobacterial DNA had been PCR-amplified and no Lineage 4 strains had been genome-sequenced. Library preparation was completed in the post-PCR laboratory. All pipettes were ultraviolet treated and all benches and equipment cleaned with hypochlorite and wiped with 80% ethanol before and after these procedures. Gloves were changed between handling different samples. Preparation of sequencing libraries for *M. bovis* Bacillus Calmette-Guérin (BCG) had been carried out previously in these laboratories, but no contamination of Illumina libraries with BCG sequences was seen in any of the intervening sequencing runs, nor in any of the libraries analysed here.

There are no plausible human or environmental sources for *M. tuberculosis* DNA in our laboratory. The presence of distinct genotypes of *M. tuberculosis* in each of the samples, aside from the mother/daughter pair, rules out cross-contamination between samples as the source of *M. tuberculosis* sequences. In the mother/daughter pair, the marked difference in the proportions of the two *M. tuberculosis* genotypes suggests cross-contamination is unlikely.

### Initial analysis of historical *M. tuberculosis* genotypes

In preliminary analyses, single MiSeq runs from three bodies (bodies 78, 80, 92) and de-multiplexed MiSeq data from other samples were analysed by mapping reads to the H37Rv genome using Bowtie2 (ref. 35), with stringent mapping parameters that specify no more than 3 mismatches per 100 bases:

--mp 1,1 --ignore-quals --score-min L,0,-0.03.

We also excluded reads that matched to rRNA genes. These stringent conditions rule out spurious matches to reads from environmental mycobacteria allowing selection of samples, which had appreciable coverage of TB genomes. Under these conditions, we obtained convincing even coverage for eight samples (Table 1) and uneven, low depth of coverage for the others (Supplementary Table 1). Aligned BAM files were analysed using MapDamage2 (ref. 36) for evidence of DNA damage associated with ancient DNA, revealing little to no C to T or G to A conversion at the ends of reads (Fig. 2).

Additional single MiSeq and HiSeq runs on the same and/or samples from the eight putatively infected mummies (Supplementary Table 1) were pooled with the original reads for genomic evaluation. The final pooled reads were filtered with Bowtie2 using the relaxed parameters:

--local -D 10 -R 3 -N 0 -L 20 -i S,1,0.50

to allow sensitive recovery of reads specific to *M. tuberculosis*. Specificity was then ensured by filtering the mapping results with the Python script post_filter.py, which retained pairs of reads where the mapped region in at least one read was ⩾100 bp in length and ⩾97.5% identical to the H37Rv reference (using a conservative 100 bp cutoff enabled improved specificity of mapping and excluded short spurious matches to sequences from related organisms). For each sample, although we also estimated the number of reads that mapped to the human genome hg19, we found relatively few reads of human origin (Supplementary Table 1).

### Phylogenetic analysis of samples from eight bodies

In an initial phylogenetic analysis, we examined all reads that mapped to H37Rv and overlapped position 3,296,371 in the H37Rv genome, which marks a seven base-pair deletion characteristic of Lineage 4 (to which H37Rv belongs)^8^,^9^. All such reads showed the same sequence as H37Rv, so we concluded that all our historical genotypes belong to Lineage 4. For detailed phylogenetic analysis of historical genotypes, we selected unassembled draft genomes from the European Nucleotide Archive Short Read Archives drawn from four sets of genotypes. These contemporary Lineage 4 genomes were derived from four distinct geographical settings: Malawi (Short Read Archive accession code ERP001072), the UK (ERP00027), Russia (ERP000192) and the Netherlands (ERP000111)^11^,^12^,^13^,^14^. From these collections, we selected unassembled draft genomes belonging to various sub-lineages of Lineage 4, based on lineage-specific IS*6110* insertions^29^ at the following positions (relative to reference strain 9177-77): 486,196 for Harlem and X clades, 935,456 for LAM clade and 893,641 for T clade. ERR234658 from the Beijing lineage was chosen as an outgroup.

Reads from the contemporary Lineage 4 unassembled draft genomes were mapped to H37Rv using Bowtie2 with default settings^2^. SNVs were called using Samtools^37^ with parameters

mpileup -AB -Q 0 -I -C20 -h 50 –gf

followed by BCFtools^11^ with parameters

view -g -cp 1

We tagged as ambiguous all sites with <20-fold coverage and where <80% of reads supported a single nucleotide. Within the entire data set, phylogenetic analyses were restricted to sites that were unambiguous in ⩾90% of the genomes and were outside of the repetitive regions in Supplementary Data 3. Genomes with >10% ambiguous bases were then removed.

We called SNVs for B68 (Terézia Hausmann) in two phases. In the first phase, we binned SNVs to the B68-1 and B68-2 genotypes by depth of coverage. As shown in supplementary Fig. 2A, the frequency of reads supporting each SNV fell into a bimodal distribution with minimal overlap, so that we could call informative SNVs by assigning those with ≤48% to B68-2 and ⩾52% to B68-1. After these procedures, eight uninformative heterogeneous sites remained unassigned and were discarded. In a second confirmatory phase, we used a phylogeny–aware approach (MGPlacer, see below) to analyse the B68 reads, using a pre-computed Lineage 4 phylogeny (see below), which gave identical results.

We confirmed that the two high-coverage unmixed historical genomes contained comparable numbers of informative SNVs to the contemporary genomes. When considering all informative sites (SNVs present in other genotypes), the proportion of uncovered sites in B80 was found to be only 1.2% (compared with an average of 0.5% sites for the modern genomes). This means that we can expect to be missing no more than one or two B80-specific SNVs. The B92-1 genome has an extremely high coverage (186X and >95% in the sample), so we see only 0.1% uncovered informative sites. As shown in Supplementary Fig. 2B, all previously known SNVs were supported in B92-1 by >85% of the reads. This means that the SNVs in B92-1 are as good as in any modern genome.

The two historical genomes derived from B68, together with two other historical draft genomes with >10-fold coverage (B80, B92-1) were then combined with the 1,582 modern genomes to create a set of 1,586 Lineage 4 genomes (plus Beijing ERR234658), which were subjected to phylogenetic analysis. A maximum-likelihood tree (Fig. 3a) was constructed from the concatenated SNVs from the 1,586 *M. tuberculosis* genomes (Supplementary Data 1) using RAxML 7.2.8 (ref. 38) with the GTRCAT model and illustrated with FigTree v1.4.2 (http://tree.bio.ed.ac.uk/software/figtree).

### MGplacer

Conventional phylogenetic methods that rely on identification of all trusted SNVs within a genome cannot be applied to the low-coverage genome sequences we obtained from most samples. We thus adapted the technique of phylogenetic placement, whereby low-coverage genomes are placed on a fixed reference tree, computed from high-coverage genomes. To do this, we devised a new algorithm, MGplacer, capable of mapping low-coverage genomes, even from samples with mixed genomes. MGplacer provides improved performance when applied to samples with mixed genotypes from the same phlogeny in a monomorphic organism like *M. tuberculosis*. When using MGplacer, the assignment of reads to the low-coverage genotypes in mixed samples relies on mapping of each read to a chain of nodes in an existing pre-computed phylogeny derived from >1,500 strains. It does not rely on depth of coverage during this process, or does it require all regions of the genome to be recovered. Supplementary Fig. 1 provides a visual of the strength of evidence for the deduced lineage mapping using this approach. The scripts for implementing MGplacer and other scripts described here for public download at https://sourceforge.net/projects/mgplacer/files/.

*Reconstructing ancestral states at all phylogenetic nodes (script MGplacer.R)*. Ancestral states for all nodes in the maximum-likelihood 1,586-Lineage 4 genome phylogeny (Fig. 3a) were determined by a time-reversible Markov process^39^, modified from the ACE function in the APE package in R^40^. This process uses the JC69 model with a fixed substitution rate, calculated as the number of all polymorphic sites divided by the total length of non-repetitive sites in the reference genome. The most likely ancestral state at each node was calculated as the maximum *a posteriori* state by the Viterbi algorithm, a dynamic programming algorithm commonly used for finding the most likely sequence of hidden states^41^.

*Branch locations of mummy genotypes with low coverage (script MGplacer2.py)*. Reads that covered polymorphic sites in the 1,586-genome phylogeny were extracted from the output of the post_filter.py script. SNVs were classified as ‘supported' when the number of supporting reads was ⩾1/3 of the median coverage of all polymorphic sites within that draft genome and otherwise as ‘not supported ‘. The likelihood of the assignment of a genotype to a branch *b* in the phylogeny was calculated from a 2 by 2 table:


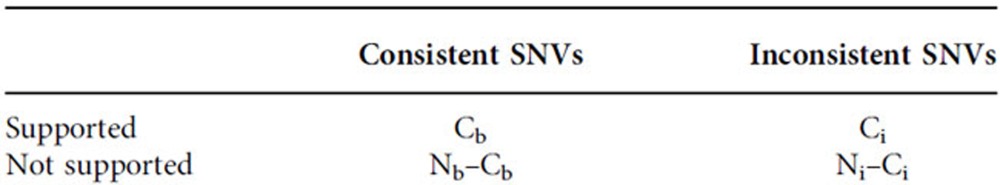


where *N*_*b*_ refers to the number of all SNVs that are consistent with a branch assignment to *b*, of which *C*_*b*_ are supported. Similarly, *N*_*i*_ is the number of all SNVs that are inconsistent with an assignment onto *b*, of which *C*_*i*_ are supported. Assuming a binomial distribution of the ratio of supported/not supported SNVs, the likelihood of a genotype belonging to branch *b* is:


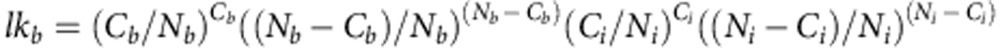


The significance (Supplementary Fig. 1) of these branch assignments was tested by the rank order of *lk*_*b*_ in 10,000 random permutations of SNV support across all branches in the tree. To detect multiple genotypes within each sample, we used MGplacer in an iterative manner, in which iterations were continued until no further significant branch assignment were achieved. After each iteration, only inconsistent SNVs and their supporting reads were retained for the subsequent iterations. Examples of the percentage of reads assigned to the major and minor genotypes in high-coverage samples are shown in Supplementary Figs. 2A and B and examples of the numbers of reads supporting each genotype in low-coverage samples in Supplementary Fig. 1A–D. Examples of low-coverage samples with no significant reads from more than one genotype are shown in Supplementary Fig. 1E and F.

### *In silico* spoligotyping

SpolPred was used to calculate the spoligotype pattern^42^ and TB-lineage used to predict the representative clade from this pattern^43^. The spoligotype was called as ‘orphan' if the probability given by TB-lineage was less than 0.8.

### Bayesian estimates of age and population fluctuation

Calculation of the root to tip distances versus dates of isolation indicated linear relationships when using only modern isolates (*R*^2^=0.063) or both historical and modern isolates (*R*^2^=0.21; Fig. 4) using Path-O-Gen (http://tree.bio.ed.ac.uk/software/pathogen/). We estimated the population history of lineage 4 with the Bayesian algorithms in Beast v1.8.0 (ref. 17). The input consisted of four historical genotypes with >10X coverage (Table 1), as well as 161 modern genomes (Supplementary Table 2), which represented the widest range of isolation dates as well as the genetic diversity in the maximum likelihood phylogeny (Supplementary Fig. 2A and Supplementary Data 2). A total of 16,449 SNVs (selected from Supplementary Data 1) in the non-repetitive core genome, supplemented by the numbers of invariant A, C, T and G nucleotides were considered in the Bayesian estimates. The dates of isolation for each strain were included in the Bayesian model as tip dates. Initial comparisons showed that the root positions of all maximum clade credibility trees differed from the root indicated by outgroup analysis in the maximum-likelihood phylogeny, which would reduce the dating accuracy of the MRCA (time to most recent common ancestor (TMRCA)). We therefore assigned lineages 4.1, 4.2 and 4.a to single monophyletic clades in order to ensure that the root was between these three lineages. A total of 12 independent Markov Chain Monte Carlo analyses were run for different combinations of clock rate and population models (Supplementary Table 4) for 100 million states, with sampling every 1,000 iterations. The initial five million samples from the beginning of each run were treated as burn-in because they had significantly lower likelihoods or priors than subsequent samples.

The Bayes factors for all 12 models were evaluated with two methods, path sampling and stepping-stone sampling, both of which are integrated in BEAST v1.8.0 and out-performed other existing methods^44^. Both methods require Markov Chain Monte Carlo sampling from a series of power posteriors. To initiate this sampling, a chain of 5M was first run as burn-in, and then 50 path steps, each of which contains 100 K burn-in and a chain length of 1M, were applied to sample the likelihood every 1,000 iterations. Of all combinations, the Bayesian model with uncorrelated lognormal distribution (UCLD) clock rates and a 30-step Bayesian Skyline population size yielded the highest Bayes factor in the path-sampling method and the second highest Bayes factor in the stepping stone method. Therefore, the samples generated from this model were applied to yield the maximum clade credibility tree and Bayesian Skyline plot in Fig. 5 and Supplementary Fig 3. Supplementary Table 3 presents the median date estimates of the best model, as well as the range of median dates from all 12 models, for the MRCA of lineage 4 and sub-lineages. Supplementary Table 4 presents the Bayes factors inferred by path sampling and stepping-stone sampling methods, as well as mutation rates and TMRCA generated from all 12 samples.

## Author contributions

M.A, H.D.D. and M.J.P. conceived the investigation. G.L.K. and J.Z.-M.C. and N.J.L. designed the experiments. I.S., I.P., M.S. and H.D.D. provided samples for analysis. G.L.K. J.Z.-M.C., J.Q. and N.J.L. performed laboratory work. G.L.K., M.J.S., Z.Z. and A.M. performed analyses. M.J.P. wrote the manuscript with contributions from all co-authors.

## Additional information

**Accession codes:** Sequence data have been deposited in the European Nucleotide Archive with the study accession code PRJEB7454.

**How to cite this article**: Kay, G. L. *et al*. Eighteenth-century genomes show that mixed infections were common at time of peak tuberculosis in Europe. *Nat. Commun.* 6:6717 doi: 10.1038/ncomms7717 (2015).

## Supplementary Material

Supplementary Figures and Supplementary Tables Supplementary Figures 1-3 and Supplementary Tables 1-4

Supplementary Data 1 SNVs assigned to branches in the phylogeny based on 1,587 TB genotypes

Supplementary Data 2 The phylogeny based on 1,587 TB genotypes in Nexus format

Supplementary Data 3 Repetitive regions in the *M. tuberculosis* H37Rv genome excluded for analysis in calling SNVs

## Figures and Tables

**Figure 1:**
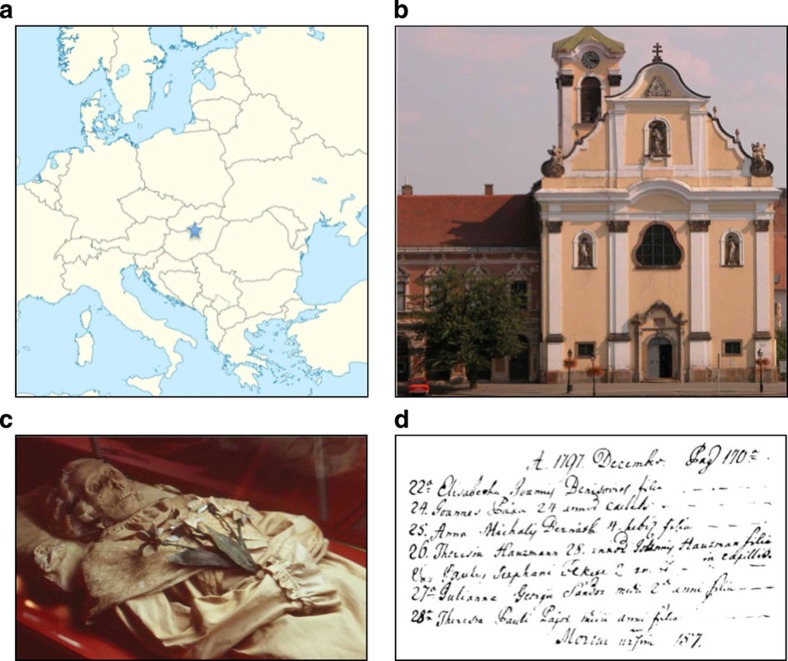
Source of eighteenth century *M. tuberculosis* genomes. (**a**) Location of Vác, Hungary. (**b**) Dominican church housing Vác mummies (©András Tumbász). (**c**) Mummified remains of Terézia Hausmann (©Hungarian Natural History Museum). (**d**) Record of Terézia Hausmann's death (©Hungarian Natural History Museum).

**Table 1:** Biographical data with sequence coverage and sub-lineages of historical *M. tuberculosis* genomes.

**Name**	**Body #**	**Sex**	**Age at death (years)**	**Date of death**	**Source of sample**	**Reads per sample (millions)**	**Reads mapping to** **H37Rv (thousands)**	**Genome**	**%**	**Coverage**	**Sub-lineage**^20^
László Beniczky	121	M	∼38	1764	Rib	48	182	B121-1	82	5.2X	4.7
								B121-2	10	0.7X	4.3
								B121-3	8	0.5X	4.b
Terézia Hausmann	68	F	28	1797	Left lung	26	11,425	B68-1*	57	332X	4.1.2.1
								B68-2†	43	253X	4.7
Anna Schneller	78	F	48	1795	Rib	17	116	B78		5.3X	4.7
Anna Schőner	28	F	55	1793	Abdomen	60	202	B28-1*	75	6.1X	4.1.2.1
								B28-2†	25	2.1X	4.7
Gáspár Steitel	25	M	58	1794	Abdomen	64	50	B25		1.7X	4.a
Erzsébet Virágh	80	F	37	1805	Thorax	14	253	B80		12.8X	4.1.2.1
Unknown	23	M	>18	1731–1838	Abdomen	42	182	B23-1	93	5.9X	4.8
								B23-2	7	0.4X	4.1.2.1
Unknown	92	M	20–39	1787	Thorax	20	4,236	B92-1	96	187X	4.8
								B92-2	4	8.6X	4.8

F, female; M, male.

%Indicates percentage of reads belonging to specific genotype of all reads that map to *M. tuberculosis* H37Rv from a mixed sample.

^*^Genotypes B28-1 and B68-1 are indistinguishable.

^†^Genotypes B28-2 and B68-2 are indistinguishable.

## References

[b1] Koch R. Die Aetiologie der Tuberkulose. Berl. Klin. Wochenschr. 19, 221–230 (1882) .

[b2] Comas I. . Out-of-Africa migration and Neolithic coexpansion of *Mycobacterium tuberculosis* with modern humans. Nature Genet. 45, 1176–1182 (2013) . 23995134 10.1038/ng.2744 PMC3800747

[b3] Bos K. I. . Pre-Columbian mycobacterial genomes reveal seals as a source of New World human tuberculosis. Nature 514, 494–497 (2014) . 25141181 10.1038/nature13591 PMC4550673

[b4] Donoghue H. D. Insights into ancient leprosy and tuberculosis using metagenomics. Trends Microbiol. 21, 448–450 (2013) . 23932433 10.1016/j.tim.2013.07.007

[b5] Chan J. Z. . Metagenomic analysis of tuberculosis in a mummy. N. Engl. J. Med. 369, 289–290 (2013) . 23863071 10.1056/NEJMc1302295

[b6] Fletcher H. A., Donoghue H. D., Holton J., Pap I. & Spigelman M. Widespread occurrence of Mycobacterium tuberculosis DNA from 18th-19th century Hungarians. Am. J. Phys. Anthropol. 120, 144–152 (2003) . 12541332 10.1002/ajpa.10114

[b7] Schuenemann V. J. . Genome-wide comparison of medieval and modern *Mycobacterium leprae*. Science 341, 179–183 (2013) . 23765279 10.1126/science.1238286

[b8] Gagneux S. . Variable host–pathogen compatibility in Mycobacterium tuberculosis. Proc. Natl Acad. Sci. USA 103, 2869–2873 (2006) . 16477032 10.1073/pnas.0511240103 PMC1413851

[b9] Marmiesse M. . Macro-array and bioinformatic analyses reveal mycobacterial 'core' genes, variation in the ESAT-6 gene family and new phylogenetic markers for the Mycobacterium tuberculosis complex. Microbiology. 150, 483–496 (2004) . 14766927 10.1099/mic.0.26662-0

[b10] Gagneux S. & Small P. M. Global phylogeography of Mycobacterium tuberculosis and implications for tuberculosis product development. Lancet Infect. Dis. 7, 328–337 (2007) . 17448936 10.1016/S1473-3099(07)70108-1

[b11] Bryant J. M. . Inferring patient to patient transmission of Mycobacterium tuberculosis from whole genome sequencing data. BMC Infect. Dis. 13, 110 (2013) . 23446317 10.1186/1471-2334-13-110 PMC3599118

[b12] Casali N. . Evolution and transmission of drug-resistant tuberculosis in a Russian population. Nature Genet. 46, 279–286 (2014) . 24464101 10.1038/ng.2878 PMC3939361

[b13] Walker T. M. . Whole-genome sequencing to delineate Mycobacterium tuberculosis outbreaks: a retrospective observational study. Lancet Infect. Dis. 13, 137–146 (2013) . 23158499 10.1016/S1473-3099(12)70277-3 PMC3556524

[b14] Coll F. . PolyTB: A genomic variation map for Mycobacterium tuberculosis. Tuberculosis (Edinb) 94, 346–354 (2014) . 24637013 10.1016/j.tube.2014.02.005 PMC4066953

[b15] Coll F. . A robust SNP barcode for typing Mycobacterium tuberculosis complex strains. Nat. Commun. 5, 4812 (2014) . 25176035 10.1038/ncomms5812 PMC4166679

[b16] Drummond A., Pybus O. G. & Rambaut A. Inference of viral evolutionary rates from molecular sequences. Adv. Parasitol. 54, 331–358 (2003) . 14711090 10.1016/s0065-308x(03)54008-8

[b17] Drummond A. J., Suchard M. A., Xie D. & Rambaut A. Bayesian phylogenetics with BEAUti and the BEAST 1.7. Mol. Biol. Evol. 29, 1969–1973 (2012) . 22367748 10.1093/molbev/mss075 PMC3408070

[b18] Muller R., Roberts C. A. & Brown T. A. Genotyping of ancient Mycobacterium tuberculosis strains reveals historic genetic diversity. Proc. Biol. Sci. 281, 20133236 (2014) . 24573854 10.1098/rspb.2013.3236 PMC3953847

[b19] Wilson L. G. Commentary: Medicine, population, and tuberculosis. Int. J. Epidemiol. 34, 521–524 (2005) . 15465901 10.1093/ije/dyh196

[b20] Newsholme A. An inquiry into the principal causes of the reduction in the death-rate from phthisis during the last forty years, with special reference to the segregation of phthisical patients in general institutions. J. Hyg. 6, 304–384 (1906) . 20474272 10.1017/s0022172400002965 PMC2236177

[b21] Drummond A. J., Rambaut A., Shapiro B. & Pybus O. G. Bayesian coalescent inference of past population dynamics from molecular sequences. Mol. Biol. Evol. 22, 1185–1192 (2005) . 15703244 10.1093/molbev/msi103

[b22] Rothschild B. M. . *Mycobacterium tuberculosis* complex DNA from an extinct bison dated 17,000 years before the present. Clin. Infect. Dis. 33, 305–311 (2001) . 11438894 10.1086/321886

[b23] Nicklisch N. . Rib lesions in skeletons from early neolithic sites in Central Germany: on the trail of tuberculosis at the onset of agriculture. Am. J. Phys. Anthropol. 149, 391–404 (2012) . 23042554 10.1002/ajpa.22137

[b24] Hershkovitz I. . Detection and molecular characterization of 9,000-year-old Mycobacterium tuberculosis from a Neolithic settlement in the Eastern Mediterranean. PLoS ONE 3, e3426 (2008) . 18923677 10.1371/journal.pone.0003426 PMC2565837

[b25] Hanekom M. . Population structure of mixed Mycobacterium tuberculosis infection is strain genotype and culture medium dependent. PLoS ONE 8, e70178 (2013) . 23936157 10.1371/journal.pone.0070178 PMC3728311

[b26] Warren R. M. . Patients with active tuberculosis often have different strains in the same sputum specimen. Am. J. Respir. Crit. Care. Med. 169, 610–614 (2004) . 14701710 10.1164/rccm.200305-714OC

[b27] Shamputa I. C. . Genotypic and phenotypic heterogeneity among Mycobacterium tuberculosis isolates from pulmonary tuberculosis patients. J. Clin. Microbiol. 42, 5528–5536 (2004) . 15583277 10.1128/JCM.42.12.5528-5536.2004 PMC535260

[b28] Hingley-Wilson S. M. Metagenomic analysis of tuberculosis--current limitations. N. Engl. J. Med. 369, 1572 (2013) . 24131193 10.1056/NEJMc1311596

[b29] Doughty E. L., Sergeant M. J., Adetifa I., Antonio M. & Pallen M. J. Culture-independent detection and characterisation of Mycobacterium tuberculosis and M. africanum in sputum samples using shotgun metagenomics on a benchtop sequencer. PeerJ 2, e585 (2014) . 25279265 10.7717/peerj.585 PMC4179564

[b30] Worby C. J., Lipsitch M. & Hanage W. P. Within-host bacterial diversity hinders accurate reconstruction of transmission networks from genomic distance data. PLoS Comput. Biol. 10, e1003549 (2014) . 24675511 10.1371/journal.pcbi.1003549 PMC3967931

[b31] Masson M. . Osteological and biomolecular evidence of a 7000-year-old case of hypertrophic pulmonary osteopathy secondary to tuberculosis from neolithic hungary. PLoS ONE 8, e78252 (2013) . 24205173 10.1371/journal.pone.0078252 PMC3813517

[b32] Kodmon C. . Molecular clues of a microepidemy among homeless tuberculosis patients in Budapest due to a new and local Mycobacterium tuberculosis clade. Infect. Genet. Evol. 7, 632–635 (2007) . 17646135 10.1016/j.meegid.2007.06.003

[b33] Pallen M. J. Diagnostic metagenomics: potential applications to bacterial, viral and parasitic infections. Parasitology 141, 1–7 (2014) . 24576467 10.1017/S0031182014000134 PMC4255322

[b34] Fletcher H. A., Donoghue H. D., Taylor G. M., van der Zanden A. G. & Spigelman M. Molecular analysis of *Mycobacterium tuberculosis* DNA from a family of 18th century Hungarians. Microbiology 149, 143–151 (2003) . 12576588 10.1099/mic.0.25961-0

[b35] Langmead B. & Salzberg S. L. Fast gapped-read alignment with Bowtie 2. Nat. Methods 9, 357–359 (2012) . 22388286 10.1038/nmeth.1923 PMC3322381

[b36] Jonsson H., Ginolhac A., Schubert M., Johnson P. L. & Orlando L. mapDamage2.0: fast approximate Bayesian estimates of ancient DNA damage parameters. Bioinformatics 29, 1682–1684 (2013) . 23613487 10.1093/bioinformatics/btt193 PMC3694634

[b37] Li H. . The Sequence Alignment/Map format and SAMtools. Bioinformatics 25, 2078–2079 (2009) . 19505943 10.1093/bioinformatics/btp352 PMC2723002

[b38] Stamatakis A. RAxML-VI-HPC: maximum likelihood-based phylogenetic analyses with thousands of taxa and mixed models. Bioinformatics 22, 2688–2690 (2006) . 16928733 10.1093/bioinformatics/btl446

[b39] Pagel M. Inferring the historical patterns of biological evolution. Nature 401, 877–884 (1999) . 10553904 10.1038/44766

[b40] Paradis E., Claude J. & Strimmer K. APE: Analyses of Phylogenetics and Evolution in R language. Bioinformatics 20, 289–290 (2004) . 14734327 10.1093/bioinformatics/btg412

[b41] Bykova N. A., Favorov A. V. & Mironov A. A. Hidden Markov models for evolution and comparative genomics analysis. PLoS ONE 8, e65012 (2013) . 23762278 10.1371/journal.pone.0065012 PMC3676395

[b42] Coll F. . SpolPred: rapid and accurate prediction of Mycobacterium tuberculosis spoligotypes from short genomic sequences. Bioinformatics 28, 2991–2993 (2012) . 23014632 10.1093/bioinformatics/bts544 PMC3496340

[b43] Shabbeer A. . TB-Lineage: an online tool for classification and analysis of strains of Mycobacterium tuberculosis complex. Infect. Genet. Evol. 12, 789–797 (2012) . 22406225 10.1016/j.meegid.2012.02.010

[b44] Baele G., Lemey P. & Vansteelandt S. Make the most of your samples: Bayes factor estimators for high-dimensional models of sequence evolution. BMC Bioinformatics 14, 85 (2013) . 23497171 10.1186/1471-2105-14-85 PMC3651733

