# Supplementary material for: Eighteenth-century genomes show that mixed infections were common at time of peak tuberculosis in Europe
Source: Nat Commun. 2015 Apr 7;6:6717. doi: 10.1038/ncomms7717 (PMC4396363; doi:10.1038/ncomms7717)
Supplement: Supplementary Figures and Supplementary Tables — Supplementary Figures 1-3 and Supplementary Tables 1-4 [file ncomms7717-s1.pdf]

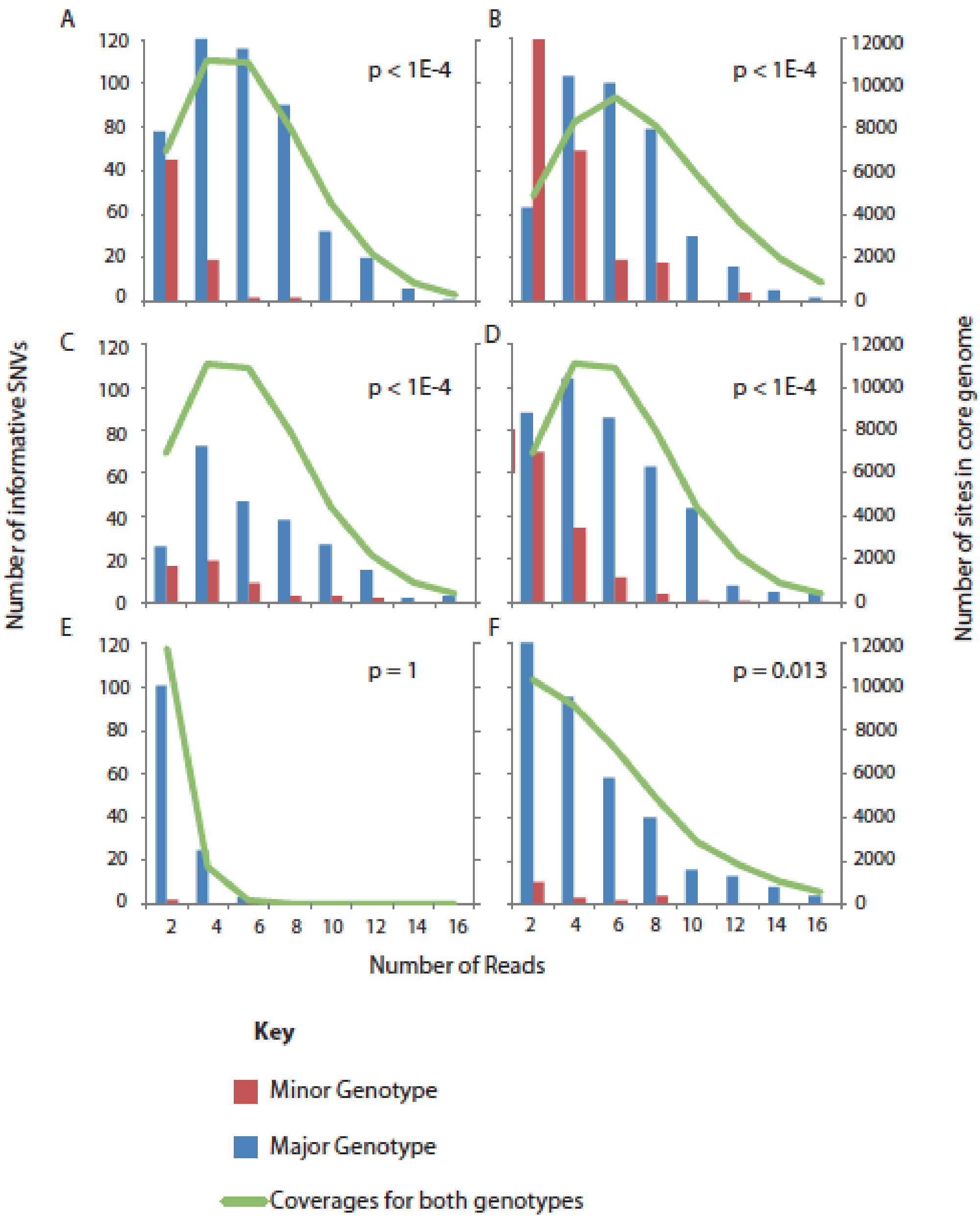

Supplementary Figure 1. Distribution of numbers of reads supporting genotype-specific SNVs according to MGplacer in low coverage samples.

Panels A-D show samples with mixed genotypes: **a.** body 23; **b.** body 28; **c.** body121, showing genotypes B121-1, and B121-2; **d.** body 121 showing genotypes B121-1 and B-121-3. Panels E and F show samples with unmixed genotypes: **e.** body 25, **f.** body 78. In each pair-wise comparison, the more common genotype is indicated in blue and the less common genotype in red. Green lines indicate the numbers of reads at all informative sites (scale at right) The significance level (p values) calculated for a minor genotype in each sample by MGplacer is shown within each graph. Although MGplacer calculated a significant p value in F, this became insignificant after Bonferroni correction for multiple samples and was not pursued further.

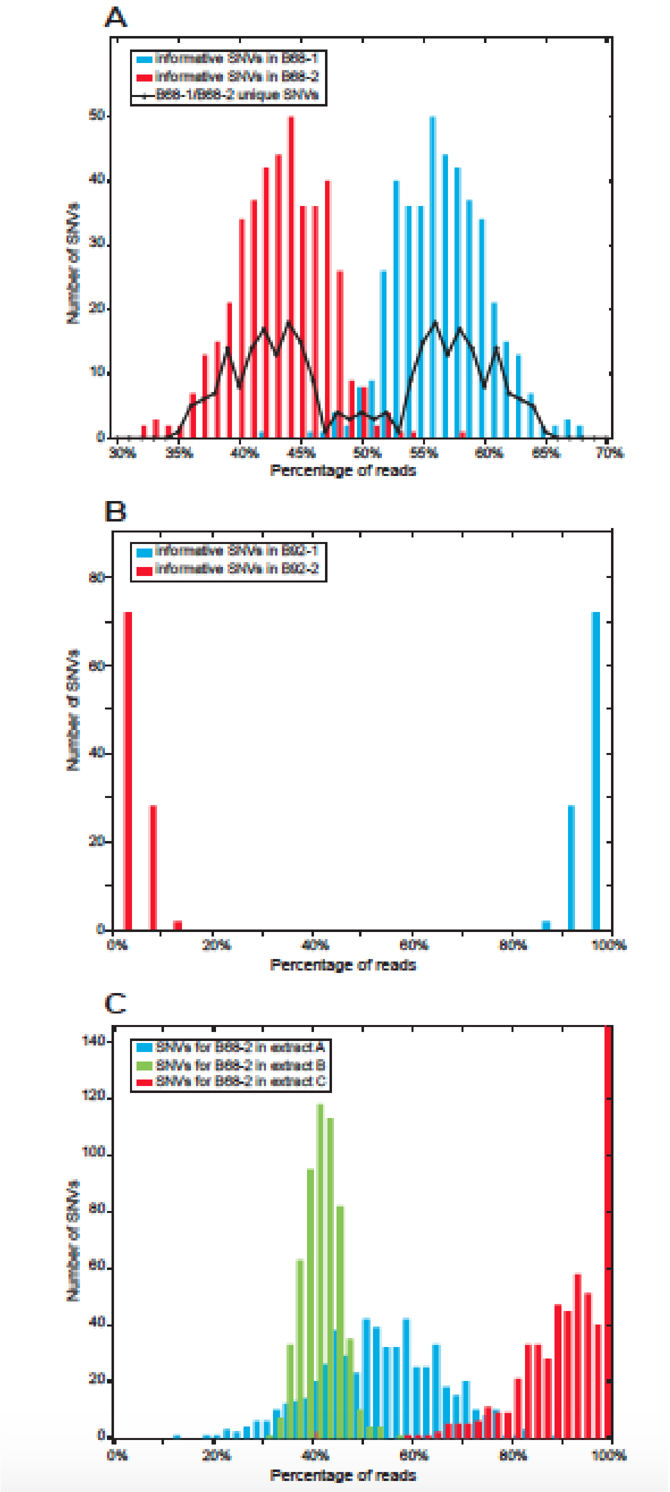

Supplementary Figure 2. Histograms of numbers of SNVs versus percentage of supporting reads in mixed samples.

a. Informative SNVs for the two body-68 genotypes shown in red and blue. Unique SNVs found in reads from this sample shown in black.

b. Informative SNVs for the two body-92 genotypes shown in red and blue

c. Percentage of reads representing the 566 SNVs that distinguish B68-2 from the other genotype, B68-1 in three separate DNA extracts. No significant differences in the frequencies of genotype-specific SNVs were found in the pairs of extractions from bodies 28 and 121.

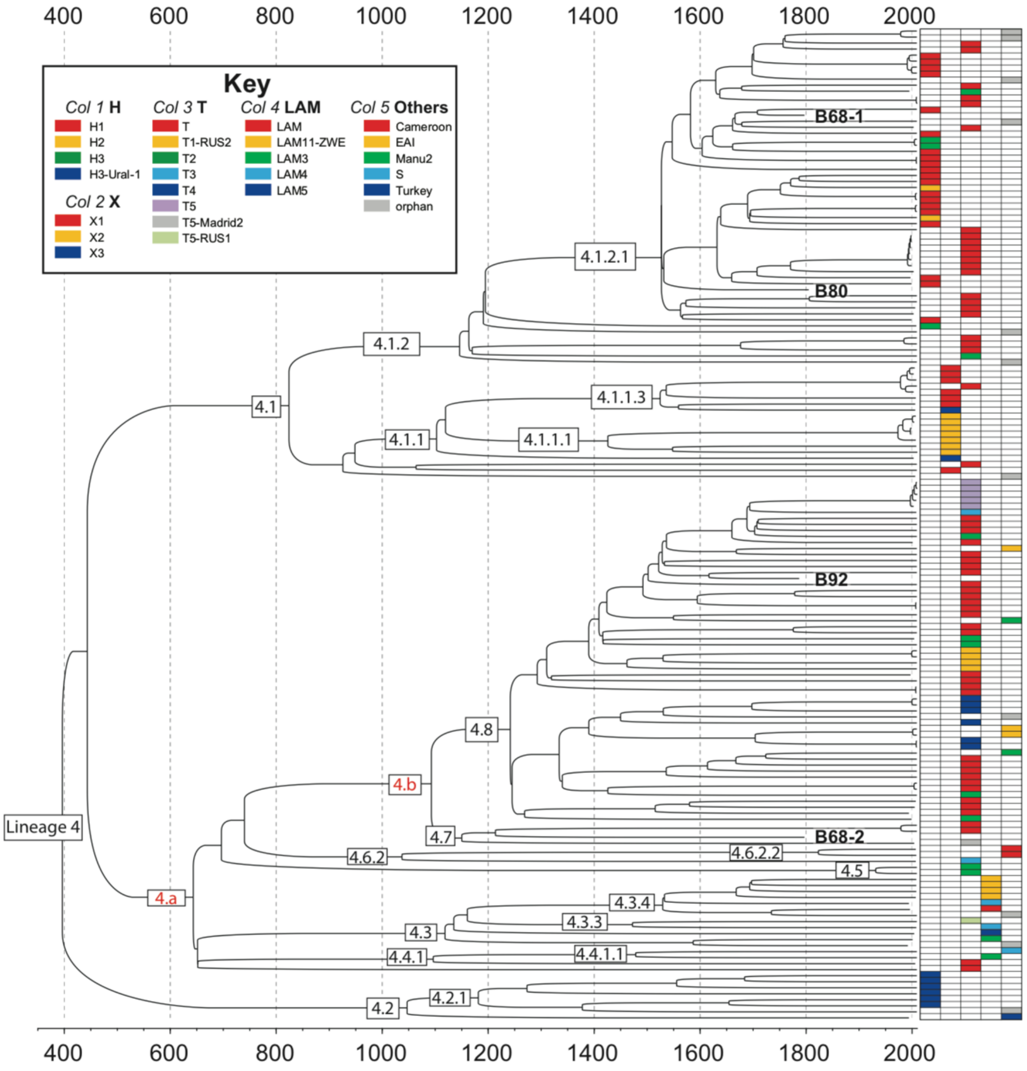

Supplementary Figure 3. Maximum clade credibility tree of 165 genotypes of Lineage 4

More detailed version of Fig. 5 with additional spoligotyping information and sub-lineages. Sub-lineages named according to Coll et al. [20] are labelled black in boxes whereas two additional lineages uncovered in our analysis (4.a and 4.b sub-lineages are labelled in red. Detailed spoligotypes of modern genomes are indicated to the right of the tree in five columns, with color-coding in the key at the upper left.

Supplementary Table 1: Biographical data and screening results on 26 bodies analysed by MiSeq and HiSeq sequencing

Body number	Name	Sex	Age at death	Date of death	Tissue sample	Platform	Paired Reads	Reads mapping to M. tb (H37Rv)	Average read length mapping to M. tb (H37Rv) (bp)	Reads mapping to Human (hg19)
4	Katalin Orlovits	F	25	5 June 1799	Chest	MiSeq	1,647,841	79	151	22
6	Mihály Orlovits	M	41	16 April 1806	Lung	MiSeq	2,843,519	115	177	120
15	Dorottya Fabó	F	66	27 Dec 1798	Right lung/pleura	MiSeq	315,5027	146	176	204
17	Vincentius Varnek	M	46	16 June 1804	Lung	MiSeq	1,256,517	159	180	873
23	Unknown	M	‘’	Unknown	Abdomen	MiSeq	1,316,550	4,819	171	255
‘’	‘’	‘’	‘’	‘’	‘’	HiSeq	40,656,814	153,478	198	9,838
24	Rozália Steitel	F	30	27 Sep 1788	Abdomen	MiSeq	1,796,192	36	179	605
‘’	‘’	‘’	‘’	‘’	‘’	HiSeq	49,073,610	1260	219	17,826
25	Chirurg Gáspár Steitel	M	58	1 Oct 1794	Abdomen	MiSeq	1,736,006	390	209	7,805
‘’	‘’	‘’	‘’	‘’	‘’	HiSeq	61,847,101	14,516	231	270,300
26	János Sikk	M	60-84	26 March 1745	Pelvic contents	MiSeq	1,735,173	92	151	84
28	Anna Schőner (mother of 68)	F	55	16 Dec 1793	Abdomen extract A	MiSeq	22,262,125	135,996	189	6,615
‘’	‘’	‘’	‘’	‘’	Abdomen extract B	MiSeq	1,757,509	1,321	191	388
‘’	‘’	‘’	‘’	‘’	‘’	HiSeq	36,081,374	28,970	212	8,979
29	József Nigrovits	M	55	11 Nov 1793	Chest extract A	MiSeq	1,494,719	7	136	2,911
‘’	‘’	‘’	‘’	‘’	Chest extract B	MiSeq	763,266	22	150	2,006
32	Terézia Waitzenbach	F	72	2 Oct 1787	Abdomen	MiSeq	447,380	7	138	3,393
‘’	‘’	‘’	‘’	‘’	Chest	MiSeq	1,580,379	47	156	490
33	György Stephanovics	M	60	12 March 1782	Stomach/intestine	MiSeq	1,123,123	24	129	6,374

37	Terézia Szigvárt (Mrs Theresia Weiskophin)	F	40	6 March 1785	Abdominal wall	MiSeq	956,226	1	186	1,614
39	József Weiskopf (Junior)	M	18	1 Aug 1785	Pelvis	MiSeq	1,317,824	4	138	1,766
‘’	‘’	‘’	‘’	‘’	Pleura	MiSeq	1,224,357	13	126	10,015
41	József Weiskopf	M	55-60	1 Jan 1785	Chest extract A	MiSeq	750,236	157	22	4,526
‘’	‘’	‘’	‘’	‘’	‘’	HiSeq	92,896,614	87	210	613,695
‘’	‘’	‘’	‘’	‘’	Chest extract B	MiSeq	653,486	6	130	65,366
44	Antal Simon	M	36	30 August 1808	Abdomen	MiSeq	2,919,980	100	170	163
48	Barbara Forstner	F	76	18 Apr 1806	Rib tissue	MiSeq	1,894,484	2	130	227
50	János Prettenhoffer	M	61	8 Feb 1806	Abdomen	MiSeq	2,466,910	63	181	681
54	Antal Nigrovits	M	29	16 July 1803	Abdomen extract A	MiSeq	1,153,043	28	172	211
‘’	‘’	‘’	‘’	‘’	Abdomen extract B	MiSeq	934,559	28	162	1635
68	Terézia Hausman (daughter of 28)	F	28	25 Dec 1797	Lung extract A	MiSeq	5,541,400	812,201	277	2,357
‘’	‘’	‘’	‘’	‘’	Lung extract B	MiSeq	19,138,733	9,403,418	236	3,114
‘’	‘’	‘’	‘’	‘’	Lung extract C	MiSeq	1,478,638	453,833	247	860
78	Anna Schneller	F	48	25 Aug 1795	Lower rib	MiSeq	17,448,828	87,706	199	1,266,208
80	Erzsébet Virágh	F	37	15 Sep 1805	Chest	MiSeq	14,425,334	175,544	272	36,188
92	Unknown	M	20-39	1787	Left chest	MiSeq	20,084,713	3,605,458	211	14173
116	Terézia Borsódi	F	26	9 Dec 1794	Pleura	MiSeq	1,588,303	31	163	427
‘’	‘’	‘’	‘’	‘’	‘’	HiSeq	136,355,165	2,404	206	36,980
120	Anna Mária Tarnóczy	F	39	30 Oct 1764	Abdomen	Miseq	1,809,166	22	191	616
121	László Beniczky	M	~38	7 Nov 1764	Rib extract A	MiSeq	14,853,214	23,096	154	6,371
‘’	‘’	‘’	‘’	‘’	Rib extract B	MiSeq	172,0137	6,492	199	445
‘’	‘’	‘’	‘’	‘’	‘’	HiSeq	31,660,130	120,946	218	6,371

Supplementary Table 2: All genotypes used in the BEAST analyses

ID	Accession code	Date	Geography	Sublineage	*in silico* Spoligotype
B68-1	This study	1797	Hungary	4.1.1.1	
B68-2	This study	1797	Hungary	4.2.1.2	
B80	This study	1805	Hungary	4.1.1.1	
B92-1	This study	1787	Hungary	4.2.1.1	
C1	ERR108463	2008-2010	Russia	4.2.1.1	T
C10	ERR133829	2008-2010	Russia	4.2	T2
C101	ERR046767	2008	Midland, UK	4.2.1.1	T5
C102	ERR046783	2008	Midland, UK	4.1.2.1	X1
C103	ERR046795	2009	Midland, UK	4.1.2.1	T
C104	ERR046821	2001	Midland, UK	4.2.1.1	T
C105	ERR046831	2007	Midland, UK	4.2.1.1	T
C106	ERR046834	2007	Midland, UK	4.2	Cameroon
C107	ERR046839	2007	Midland, UK	4.2	T3
C108	ERR046843	2005	Midland, UK	4.1.2.1	X1
C11	ERR133833	2008-2010	Russia	4.1.2	T
C112	ERR046865	2006	Midland, UK	4.1.2	orphan
C113	ERR046868	2007	Midland, UK	4.1.2.1	X1
C114	ERR046872	2003	Midland, UK	4.1.1.1	T
C115	ERR046874	2003	Midland, UK	4.1.1.1	T
C116	ERR046888	2004	Midland, UK	4.2.1.1	T2
C117	ERR046917	2003	Midland, UK	4.1.2.1	X1
C12	ERR133835	2008-2010	Russia	4.2.1.1	T
C120	ERR046928	2009	Midland, UK	4.2.1.1	T5
C121	ERR046937	2006	Midland, UK	4.1.1.1	T
C122	ERR046946	2010	Midland, UK	4.1.2.2	X2
C125	ERR046953	2008	Midland, UK	4.2.1.1	T
C127	ERR046958	2009	Midland, UK	4.1.1.1	T
C128	ERR046960	1998	Midland, UK	4.1.2.2	X2
C13	ERR133836	2008-2010	Russia	4.2.1.1	T
C130	ERR046984	2006	Midland, UK	4.1.2.1	X1
C131	ERR046986	2004	Midland, UK	4.1.2.2	X2
C133	ERR046993	2010	Midland, UK	4.1.1.1	T
C134	ERR046996	2008	Midland, UK	4.2	Cameroon
C135	ERR047000	2006	Midland, UK	4.1.2.1	X3
C137	ERR047006	2008	Midland, UK	4.2.1.1	T
C138	ERR047008	2008	Midland, UK	4.2.1.1	T
C139	ERR067579	2008-2010	Russia	4.2.1.1	T
C14	ERR133857	2008-2010	Russia	4.2.1.2	T
C141	ERR067602	2008-2010	Russia	4.2.1.1	EAI
C142	ERR067603	2008-2010	Russia	4.1.1.1	H1

C143	ERR067606	2008-2010	Russia	4.3	H3-Ural-1
C144	ERR067616	2008-2010	Russia	4.1.1.1	H2
C145	ERR067617	2008-2010	Russia	4.1.1.1	H1
C146	ERR067670	2008-2010	Russia	4.1.1.1	H1
C147	ERR067677	2008-2010	Russia	4.3	H3-Ural-1
C148	ERR067682	2008-2010	Russia	4.2.1.1	T4
C149	ERR067703	2008-2010	Russia	4.1.1	T
C15	ERR133870	2008-2010	Russia	4.1.1	T2
C150	ERR067726	2008-2010	Russia	4.2.1.1	T
C151	ERR067756	2008-2010	Russia	4.2.1.1	T4
C154	ERR072045	2010	Midland, UK	4.2.1.1	T5
C156	ERR072051	2010	Midland, UK	4.2.1.1	T5
C159	ERR108437	2008-2010	Russia	4.3	H3-Ural-1
C16	ERR133878	2008-2010	Russia	4.1.1.1	H1
C160	ERR108444	2008-2010	Russia	4.1.1	T
C161	ERR108448	2008-2010	Russia	4.2.1.1	T
C162	ERR108449	2008-2010	Russia	4.1.1.1	T
C163	ERR108456	2008-2010	Russia	4.3	H3-Ural-1
C164	ERR108457	2008-2010	Russia	4.2.1.1	T2
C165	ERR108459	2008-2010	Russia	4.1.1.1	H1
C166	ERR108461	2008-2010	Russia	4.1.1.1	H1
C167	ERR046884	2008	Midland, UK	4.2.2	LAM11-ZWE
C168	ERR046870	2008	Midland, UK	4.2.2	LAM11-ZWE
C169	ERR039330	2008	Midland, UK	4.2.2	LAM11-ZWE
C17	ERR133881	2008-2010	Russia	4.2.1.1	T
C170	ERR046836	2007	Midland, UK	4.2.2	LAM11-ZWE
C171	ERR133872	2008-2010	Russia	4.2.2	orphan
C172	ERR257910	1994	Netherland	4.2.2	LAM4
C173	ERR028629	1996	Netherland	4.2.2	LAM
C174	ERR025453	1992	Netherland	4.2.2	orphan
C175	ERR024343	2004	Netherland	4.2.2	LAM5
C176	ERR024345	2001	Netherland	4.2.2	LAM3
C177	ERR047009	2008	Midland, UK	4.2.1.1	T3
C178	ERR133864	2008-2010	Russia	4.2.1.1	T
C179	ERR228035	2008-2010	Russia	4.2.1.1	T
C18	ERR133883	2008-2010	Russia	4.1.1.1	T
C180	ERR144592	2008-2010	Russia	4.2.1.1	T
C181	ERR108427	2008-2010	Russia	4.2.1.1	T1-RUS2
C182	ERR133813	2008-2010	Russia	4.2.1.1	T1-RUS2
C183	ERR257893	1994	Netherland	4.2.1.1	T
C184	ERR257909	2004	Netherland	4.2.1.2	T5-Madrid2
C185	ERR023746	1999	Netherland	4.1.1.1	H1

C186	ERR025415	2008	Netherland	4.1.1	H3
C187	ERR046735	2007	Midland, UK	4.1.2.2	X2
C188	ERR403265	2008-2010	Russia	4.2.1.1	T
C189	ERR257900	1997	Netherland	4.2.1.1	T
C19	ERR133897	2008-2010	Russia	4.2	T
C2	ERR108474	2008-2010	Russia	4.1.1.1	H1
C20	ERR133909	2008-2010	Russia	4.2.1.1	T2
C21	ERR133938	2008-2010	Russia	4.2.1.1	T
C22	ERR133961	2008-2010	Russia	4.1.1.1	H3
C23	ERR133965	2008-2010	Russia	4.1.1.1	H1
C24	ERR133971	2008-2010	Russia	4.1.1.1	H1
C25	ERR133974	2008-2010	Russia	4.2	S
C26	ERR133980	2008-2010	Russia	4.2.1.1	T
C27	ERR133981	2008-2010	Russia	4.3	orphan
C28	ERR137191	2008-2010	Russia	4.2.1.1	Manu2
C29	ERR137197	2008-2010	Russia	4.2.1.1	T
C3	ERR108478	2008-2010	Russia	4.2.1.1	T
C30	ERR137202	2008-2010	Russia	4.1.1.1	T
C31	ERR137213	2008-2010	Russia	4.2.1.1	T4
C32	ERR137214	2008-2010	Russia	4.3	H3-Ural-1
C33	ERR137228	2008-2010	Russia	4.1.1.1	H1
C34	ERR137256	2008-2010	Russia	4.1.1.1	H1
C35	ERR137261	2008-2010	Russia	4.2.1.1	orphan
C36	ERR137266	2008-2010	Russia	4.1.1.1	orphan
C37	ERR144546	2008-2010	Russia	4.2.1.2	T
C38	ERR144559	2008-2010	Russia	4.1.1.1	H3
C39	ERR144585	2008-2010	Russia	4.2.1.1	T
C4	ERR108482	2008-2010	Russia	4.1.1.1	H1
C40	ERR144629	2008-2010	Russia	4.2.1.1	Manu2
C41	ERR158571	2008-2010	Russia	4.2.1.1	T1-RUS2
C42	ERR158599	2008-2010	Russia	4.1.1	orphan
C45	ERR228000	2008-2010	Russia	4.2.1.1	EAI
C46	ERR228021	2008-2010	Russia	4.1.1	orphan
C47	ERR228038	2008-2010	Russia	4.1.1.1	H2
C48	ERR228044	2008-2010	Russia	4.1.1.1	T
C49	ERR228045	2008-2010	Russia	4.2	T2
C5	ERR133800	2008-2010	Russia	4.1.1.1	H1
C50	ERR228047	2008-2010	Russia	4.2.1.1	EAI
C51	ERR228057	2008-2010	Russia	4.2.1.1	T
C52	ERR228067	2008-2010	Russia	4.2	LAM3
C53	ERR229925	2008-2010	Russia	4.1.1.1	H1
C54	ERR229932	2008-2010	Russia	4.1.1.1	H1
C55	ERR229938	2008-2010	Russia	4.1.1.1	T
C56	ERR229941	2008-2010	Russia	4.1.1.1	orphan
C57	ERR229944	2008-2010	Russia	4.2.2	T5-RUS1

C58	ERR229948	2008-2010	Russia	4.2.1.1	T4
C59	ERR229950	2008-2010	Russia	4.1.1.1	H1
C6	ERR133807	2008-2010	Russia	4.2.1.1	T
C60	ERR229954	2008-2010	Russia	4.1.1.1	T
C61	ERR229956	2008-2010	Russia	4.2.1.1	T4
C62	ERR229963	2008-2010	Russia	4.2.1.1	T
C63	ERR229990	2008-2010	Russia	4.2.1.1	T
C64	ERR230001	2008-2010	Russia	4.2.1.1	T2
C65	ERR234580	2008-2010	Russia	4.1.1.1	orphan
C66	ERR234592	2008-2010	Russia	4.2.1.1	T1-RUS2
C67	ERR234627	2008-2010	Russia	4.1.2	X1
C69	ERR234636	2008-2010	Russia	4.1.1	T
C7	ERR133818	2008-2010	Russia	4.1.1.1	orphan
C71	ERR234684	2008-2010	Russia	4.1.1.1	H1
C72	ERR257897	1997	Netherland	4.1.1.1	H1
C74	ERR257932	1996	Netherland	4.1.1.1	T
C75	ERR257933	1999	Netherland	4.1.1.1	T
C76	ERR257934	1999	Netherland	4.1.1.1	T
C77	ERR257935	1999	Netherland	4.1.1.1	T
C78	ERR025429	1999	Netherland	4.1.1.1	T
C8	ERR133823	2008-2010	Russia	4.3	H3-Ural-1
C80	ERR026475	1999	Netherland	4.2.1.1	T2
C81	ERR028612	1998	Netherland	4.1.1.1	T
C82	ERR028616	1994	Netherland	4.3	Turkey
C83	ERR028624	1995	Netherland	4.1.1.1	T2
C84	ERR028626	1999	Netherland	4.1.1.1	H1
C85	ERR038276	2003	Midland, UK	4.1.2.1	X1
C86	ERR038278	2003	Midland, UK	4.1.2	X3
C87	ERR038290	2008	Midland, UK	4.2.2	LAM4
C88	ERR039326	2008	Midland, UK	4.1.2.2	X2
C89	ERR039328	2007	Midland, UK	4.1.2.2	X2
C9	ERR133828	2008-2010	Russia	4.2.1.1	T4
C90	ERR039339	2008	Midland, UK	4.2.1.1	T
C91	ERR039342	2005	Midland, UK	4.2.1.1	T
C92	ERR039343	2006	Midland, UK	4.2	T
C93	ERR039344	2003	Midland, UK	4.1.1.1	T
C95	ERR040105	2003	Midland, UK	4.2.1.1	T5
C96	ERR046738	1999	Midland, UK	4.1.2.2	X2

Supplementary Table 3: *M. tuberculosis* Lineage 4 and its sub-lineages: dates and lineage-specific features

Lineage	Median date of most recent common ancestor	Date ranges	Alternative epidemiological designations	Lineage-specific # SNVs (Dataset 1)
4	396 CE	1273 BCE - 439 CE	Euro-American; EAM	-
4.1	824 CE	47 BCE - 853 CE		78
4.1.1	1103 CE	731 CE - 1124 CE	X	33
4.1.1.1	1426 CE	1189 CE - 1446 CE	X1	53
4.1.1.3	1525 CE	1241 CE - 1539 CE	X2	98
4.1.2	1146 CE	462 CE - 1187 CE		15
4.1.2.1	1527 CE	907 CE - 1552 CE	Haarlem	65
4.2	1047 CE	325 CE - 1081 CE		147
4.3	1119 CE	600 CE - 1150 CE	LAM	105
4.a	643 CE	279 BCE - 679 CE		47
4.b	1093 CE	375 CE - 1124 CE	T, PCG3	43
4.7	1150 CE	804 CE - 1181 CE		11
4.8	1242 CE	566 CE - 1271 CE		20

Supplementary Table 4: Comparison of twelve Bayesian models in BEAST analyses.

Model		Model evaluation		Parameters	
Clock	Population	PS	SS	Median TMRCA (CI95%)	Median Mutation Rate (CI95%)
UCLD	Bayesian Skyline (30)	-6096937	-6096177	396 CE (40 CE - 662 CE)	5.00E-08 (4.06 - 5.87E-08)
Constant	Bayesian Skyline (10)	-6096943	-6096178	439 CE (146 CE - 663 CE)	5.14E-08 (4.32 - 5.91E-08)
UCLD	EBSP	-6096948	-6096188	383 CE (63 CE - 651 CE)	4.98E-08 (4.11 - 5.82E-08)
Constant	Bayesian Skyline (30)	-6096969	-6096194	423 CE (115 CE - 662 CE)	5.08E-08 (4.22 - 5.89E-08)
Constant	EBSP	-6096971	-6096197	411 CE (109 CE - 654 CE)	5.06E-08 (4.24 - 5.89E-08)
UCED	Bayesian Skyline (10)	-6097022	-6096351	1085 BCE (15455 BCE - 1118 CE)	3.10E-08 (0.21 - 5.83E-08)
UCLD	Bayesian Skyline (10)	-6097083	-6096170	424 CE (104 CE - 684 CE)	5.10E-08 (4.19 - 5.95E-08)
UCED	Bayesian Skyline (30)	-6097205	-6096357	1273 BCE (11284 BCE - 984 CE)	2.86E-08 (0.43 - 5.45E-08)
UCED	EBSP	-6097207	-6096358	810 BCE (14188 BCE - 933 CE)	2.70E-08 (0.42 - 3.12E-08)
UCLD	Constant	-6097243	-6096285	414 CE (99 CE - 664 CE)	5.13E-08 (4.23 - 5.96E-08)
Constant	Constant	-6097454	-6096285	438 CE (168 CE - 676 CE)	5.19E-08 (4.37 - 5.97E-08)
UCED	Constant	-6097562	-6096417	585 BCE (13933 BCE - 965 CE)	2.92E-08 (0.31 - 3.83E-08)

Note: Models are ordered from top to bottom (best to worst) according to decreasing Bayes factors based on path sampling (PS); SS – stepping-stone sampling; UCLD - uncorrelated lognormal distribution; UCED - uncorrelated exponential distribution; EBSP - Extended Bayesian Skyline Plot;
